# Modulating NLRP3 Inflammasomes in Idiopathic Pulmonary Fibrosis: A Comprehensive Review on Flavonoid-Based Interventions

**DOI:** 10.1007/s12013-025-01696-4

**Published:** 2025-02-19

**Authors:** Megh Pravin Vithalkar, Shreya Pradhan, K. S. Sandra, H. B. Bharath, Yogendra Nayak

**Affiliations:** Department of Pharmacology, Manipal College of Pharmaceutical Sciences, Manipal Academy of Higher Education, Manipal, Karnataka Pin 576104 India

**Keywords:** Idiopathic Pulmonary Fibrosis, Extracellular matrix, NLRP3 inflammasome, Flavonoids, PANoptosis

## Abstract

Idiopathic Pulmonary Fibrosis (IPF) is a severe, rapidly advancing disease that drastically diminishes life expectancy. Without treatment, it can progress to lung cancer. The precise etiology of IPF remains unknown, but inflammation and damage to the alveolar epithelium are widely thought to be pivotal in its development. Research has indicated that activating the NLRP3 inflammasome is a crucial mechanism in IPF pathogenesis, as it triggers the release of pro-inflammatory cytokines such as IL-1β, IL-18, and TGF-β. These cytokines contribute to the myofibroblast differentiation and extracellular matrix (ECM) accumulation. Currently, treatment options for IPF are limited. Only two FDA-approved medications, pirfenidone and nintedanib, are available. While these drugs can decelerate disease progression, they come with a range of side effects and do not cure the disease. Additional treatment strategies primarily involve supportive care and therapy. Emerging research has highlighted that numerous flavonoids derived from traditional medicines can inhibit the critical regulators responsible for activating the NLRP3 inflammasome. These flavonoids show promise as potential therapeutic agents for managing IPF, offering a new avenue for treatment that targets the core inflammatory processes of this debilitating condition.

**Graphical Abstract:**

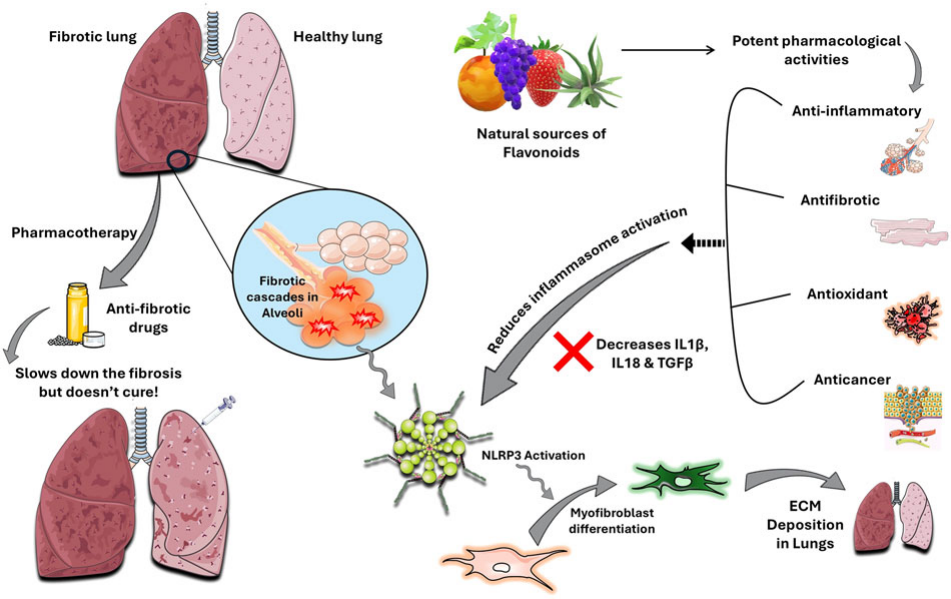

## Introduction

Idiopathic pulmonary fibrosis, also known as IPF, is a debilitating disorder that progressively causes inflammation in the lungs and leads to fibrosis. Unfortunately, its survival rate is typically only 2–5 years [1], and after the COVID-19 pandemic, the disease prevalence has risen to 44.9% [2]. IPF is marked by the extreme extracellular matrix (ECM) buildup and alveolar tissue remodeling, forming scar tissue [3]. For those with IPF, symptoms may include non-specific issues such as difficulty breathing, dry cough, and occasionally flu-like symptoms. The progression of IPF is driven by a mix of genetic, age-related, and environmental factors [1, 3], although the exact mechanisms are still not fully understood. These factors damage the epithelial cells and cause abnormal activation, resulting in the secretion of inflammatory cytokines like TGF-β. These cytokines promote fibroblasts’ migration, proliferation, and differentiation into myofibroblasts (MYFs) [4]. MYFs then secrete a large amount of ECM, leading to the abnormal deposition of ECM and stiffness of the alveolar matrix [5]. These processes cause lung structure remodeling and the formation of fibrotic lung disease, and if untreated, it may lead to lung cancer, especially non-small cell lung cancer (NSCLC) [6].

The alveoli in the lungs comprise two types of cells called pneumocytes. The flat and squamous cells are alveolar epithelial cells (AEC)-type 1 (AEC1), whereas cuboidal cells with microvilli are AEC-type 2 (AEC2) [7]. AEC2 cells can transform into both AEC1 and AEC2. Any harm to the alveolar lining triggers the multiplication of fibroblasts mediated by AEC2, which further transforms into mesenchymal MYFs [7]. MYFs undergo apoptosis after the repair process, which restores the regular functioning of the lungs. However, in the diseased state (IPF), the normal apoptosis process is severely affected [8]. There is a discrepancy between the production and lysis of MYFs, leading to surplus collagen deposition. This causes ventilation concerns and creates an imbalance of oxygen supply in the body [9].

Abnormal gene expression within AECs is believed to be a major contributor to IPF, with epigenetic mutations playing a significant role. The MUC5B promoter region is a vital genetic risk factor for IPF [10], with polymorphism being the most replicated mutation. Other mutations, such as those found in telomerase-related genes (TERT and TERC) and surfactant protein genes (SFTPA2 and SFTPC), have also been observed in IPF patients [11]. Growth factors like PDGF, TGF-β, FGF, and VEGF act through multiple signaling pathways (WNT-Catenin, Sonic Hedgehog, TGFβ1-SMAD, and Notch), which play a vital role in the interaction between AEC and MYFs responsible for the development of fibrotic conditions [9].

Currently, there is no known cure for IPF; however, the management involves both pharmacotherapy and other supportive measures such as managing cough and dyspnoea, oxygen therapy, and ventilation, and it can be a lung transplant at the end stage of the disease [12]. The US FDA-approved drugs used in pharmacotherapy are pirfenidone and nintedanib. Pirfenidone is an anti-inflammatory agent suppressing TGF-β1-mediated fibroblast activity and collagen production [13]. On the other hand, a tyrosine kinase inhibitor, called nintedanib, primarily targets VEGF, FGF, and PDGF receptors and reduces ECM deposition and fibroblast proliferation [9, 14]. These medications only slow down the disease progression and alleviate symptoms. Hence, promising novel treatment strategies and therapeutic compounds are the need of the hour.

Several therapeutic agents are being evaluated in clinical trials for treating fibrotic diseases by targeting specific pathways and mechanisms. Tralokinumab targets IL-13 and reduces the expression of TGF-β and macrophage CCL2 (NCT01969409). Lebrikizumab is in clinical trials (NCT01872689). Simtuzumab targets LOXL2 and decreases extracellular matrix cross-linking (NCT01769196). STX-100 targets Integrin αvβ6 and decreases the activation of latent TGF-β (NCT01371305). Rituximab targets CD20 and decreases the contribution of antibody-mediated autoimmunity (NCT01969409). Carbon Monoxide has anti-inflammatory effects and may suppress fibroblast proliferation (NCT01214187). Azithromycin acts as both an antimicrobial and immunomodulatory agent (NCT02173145). Cotrimoxazole is an antimicrobial agent against *Pneumocystis jiroveci* and bacteria (NCT01777737). These agents are being evaluated in various clinical trials to determine their effectiveness in managing IPF through diverse mechanisms of action.

**Fig. 1:**
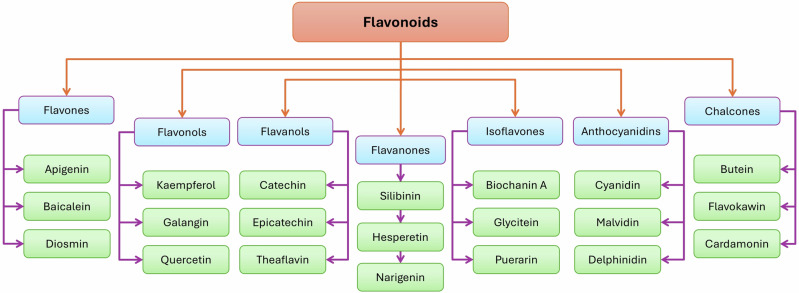
Structure-based classification and examples of Flavonoids

Academic institutions have also made significant contributions to the understanding and treatment of lung fibrosis. The University of Illinois College of Medicine at the Center for Lung and Vascular Biology, Chicago, has patented nanoparticles of TGF-β1-siRNA for targeting the mannose receptor CD206 in interstitial macrophages [15]. In the University of Edinburgh, the Queen’s Medical Research Institute developed inhalational TD139 in Phase-III trials [16]. Another study on Traditional Chinese Medicine (TCM) at Chengdu University identified 6-Gingerol as a protective agent against bleomycin (BLM)-induced pulmonary fibrosis (BIPF) in mice by activating SIRT-1 [17]. In treating IPF, Chinese traditional medicines make the most contribution compared to other traditional medicines. Hence, potent phytochemicals or small molecules from these traditional medicines are being explored further to develop into drugs [18]. Among these phytochemicals, flavonoids, a class of multiple polyphenols (Fig. 1), are highly beneficial as they show antifibrotic activities in IPF [19, 20]. These flavonoids exert their effects through several molecular pathways, including inflammasomes, focusing on the NLRP3 (nucleotide-binding domain, leucine-rich repeat, and pyrin domain-containing protein-3) pathway [21]. This review consolidates recent investigations emphasizing the therapeutic properties of natural flavonoids in IPF, specifically through the NLRP3 mechanism.

## Methodology

### Database Selection and Search Strategy

The literature review for this comprehensive narrative review was conducted using major scientific databases, including Scopus (https://www.scopus.com/), PubMed (https://pubmed.ncbi.nlm.nih.gov/), Web of Science (https://www.webofscience.com/), and Google Scholar (https://scholar.google.com/), to ensure thorough coverage of relevant studies. The search strategy incorporated keywords such as “flavonoids (specific names of the flavonoids),” “polyphenols,” “antifibrotic,” “pulmonary fibrosis,” “lung fibrosis,” “NLRP3,” and “inflammasomes,” with Boolean operators (AND, OR) to refine and optimize the search results.

### Criteria for Inclusion and Exclusion

Articles were incorporated if they explored the antifibrotic effects of flavonoids, investigated molecular mechanisms of diseases related to NLRP3 inflammasomes, and were published in peer-reviewed journals within the last ten years (80% of the articles). Articles were excluded if they did not focus on IPF, NLRP3 inflammasomes, flavonoids or centered on flavonoids in non-respiratory diseases, were not published in English, or lacked sufficient information.

### Screening and Data Extraction

The screening process involved an initial review of titles and abstracts for relevance, followed by a full-text review of articles that met the inclusion criteria. Relevant results were then extracted from the selected studies, including the types of flavonoids studied, their molecular targets (with a specific focus on NLRP3 inflammasomes), the cell lines and animal models used, and key findings related to their antifibrotic properties and mechanisms of action. Finally, the analyzed data were merged to provide a comprehensive review of the therapeutic potential of various potent flavonoids in pulmonary fibrosis, emphasizing their action through the NLRP3 inflammasome pathway.

## NLRP3 Inflammasomes

Our body naturally uses inflammation as a defense mechanism against harmful agents, whether they originate from within or outside the body. The cells involved in innate immunity can identify different danger signals, such as Pathogen-associated molecular patterns (PAMPS) created by invading microbes or Danger-associated molecular patterns (DAMPS) generated because of metabolic pathways and endogenous stress and inflammation [22]. Pattern Recognition Receptors (PRR) are crucial in detecting these PAMPS and DAMPS [23]. PRRs are classified based on location, with membrane-bound PRRs like C-type lectin receptors (CLR) and Toll-like receptors (TLR) being particularly important. PRRs in the cytoplasm include RIG-I-like receptors (RLR) and Nucleotide-binding oligomerization domain-like receptors (NLR). Once the TLRs identify PAMPs and DAMPs, they pass the responsibility to NLRs, which activate a cascade of pathways leading to the configuration of multiprotein complexes known as inflammasomes [24].

### Structure of NLRP3 Inflammasomes

The NLRP3 inflammasome comprises three components: the NLRP3 sensor protein, the apoptosis-associated spec-like protein with caspase recruitment domain (ASC), and procaspase-1. The NLRP3 sensor protein is divided into three regions: a Pyrin domain (PYD), a NACHT domain, which includes a nucleotide-binding and oligomerization domain, and a Leucin-rich repeat domain (LRR) [25]. The NACHT domain also has several sub-regions, such as the helical domains 1 and 2 (HD1 and HD2), nucleotide-binding domain (NBD), and a winged helix domain (WHD) located between the two helical domains. Additionally, the NACHT domain contains a walker A motif and a walker B motif. The former is for ATP binding and the latter for ATPase activity [26].

### Activation of NLRP3 and its Role in Inflammation

The process of assembling and activating NLRP3 is closely regulated by inflammasomes, which are responsible for inflammation and can be activated by a variety of factors such as pathogens, including yeast, bacteria, and viruses, endogenous danger signals like ATP, and particulates such as uric acid crystals, fibrillar amyloid-β, and malarial hemozoin, and environmental irritants like silica, asbestos, UVB radiation, and skin irritants. These factors can cause damage to the cell walls, leading to oxidative stress, which can also contribute to the activation of inflammasomes [27]. Activating NLRP3 involves a two-phase process, initiating with priming and then activation. The signal called priming turns on a surge in the expression of NLRP3. This protein is believed to be insufficient to activate the inflammasome during normal conditions. Additionally, pro-IL-1β is not inherently synthesized in resting macrophages [28]. The inflammasome complex requires an activation signal for final assembly. This activation signal involves relocating NLRP3 to mitochondria, which releases mitochondrial ROS, DNA, or cardiolipin. K^+^ efflux occurs through ion channels, there is lysosomal dysfunction, and cathepsin is released. These factors alone or in synergy with others can act as a triggering signal for the assembly step. There are three pathways for activating NLRP3 inflammasomes: canonical, non-canonical, and alternative.

#### Canonical pathway

The canonical pathway of NLRP3 inflammasome activation is initiated in two main stages: priming and activation [29]. The priming step is triggered by the detection of pathogen-associated molecular patterns (PAMPs) or damage-associated molecular patterns (DAMPs) via Pattern Recognition Receptors (PRRs) such as Toll-like Receptors (TLRs) or NOD-like Receptors (NLRs). These receptors recognize microbial components like lipopolysaccharide (LPS) and endogenous molecules like ATP or uric acid, signaling the activation of the NF-κB pathway [29]. The activation of NF-κB leads to the transcriptional upregulation of key inflammasome components, including NLRP3 (the inflammasome sensor) and pro-IL-1β (the precursor of the pro-inflammatory cytokine interleukin-1β). This transcriptional priming prepares the cell for the subsequent activation phase, where the inflammasome will be assembled. During this stage, various receptors such as TLRs, NOD-like receptors, and TNF receptors are activated to send signals that alert the immune system to infection or injury [30, 31]. These signals trigger an increase in the production of inflammasome components, including NLRP3, procaspase-1, and pro-IL-1β [32]. The activation phase is triggered by a variety of stimuli such as pore-forming bacterial toxins, extracellular ATP, or particulate matter like uric acid crystals, silica, and asbestos. These stimuli lead to multiple cellular events that culminate in the assembly of the NLRP3 inflammasome. A critical step in the activation process is the alteration in ion fluxes, particularly the efflux of K+, which is a dominant event in NLRP3 activation. Other ion mobilizations, including Na+, Cl−, and Ca2+, also contribute to inflammasome assembly. Additionally, mitochondrial dysfunction and the release of mitochondrial reactive oxygen species (mtROS), oxidized mitochondrial DNA (mtDNA), and cardiolipin further enhance NLRP3 activation [33]. Lysosomal disruption, triggered by particulate matter, can also induce the release of enzymes like cathepsin B, which act as additional DAMPs, promoting inflammasome activation [23].

Once activated, the next step involves the binding of NLRP3 with NEK7 and ATP, triggering conformational changes that lead to the formation of the NLRP3 inflammasome multiprotein complex [33, 34]. This complex includes ASC (apoptosis-associated speck-like protein containing a CARD) and procaspase-1, which is cleaved to caspase-1. Once activated, caspase-1 processes pro-IL-1β and pro-IL-18 into their mature, active forms, which are secreted to initiate the inflammatory response. Additionally, caspase-1 cleaves Gasdermin D (GSDMD), leading to the formation of pores in the cell membrane and resulting in pyroptosis, a form of programmed cell death. This cell death releases intracellular contents, amplifying the immune response and facilitating the recruitment of additional immune cells to the site of infection or injury. The canonical pathway for NLRP3 inflammasome activation, which involves these key steps of priming, activation, and cleavage, plays a critical role in immune responses to infection, tissue injury, and inflammatory diseases [29, 33].

#### Non-canonical pathway

This type of pathway does not depend on TLR4 signaling, therefore, it does not need a priming signal and is usually observed in gram-negative bacterial infections [23, 29]. The lipopolysaccharide (LPS) produced by the gram-negative bacteria enters the cell without going through the membrane PRRs and directly activates the caspase-4/5 in humans [35]. These caspases then cause pyroptosis by processing GSDMD and pannexin-1, forming pores in a protein channel and the cell membrane that releases ATP from the cell [36]. This pore formation aids the release of interleukins (IL-18 and IL-1β), amplifying the inflammatory reaction. The ATP then triggers an ATP-gated cation-selective channel, P2X7-receptor (P2X7R), that opens a pore and initiates K^+^ efflux. This inflammasome initiation leads to the formation of inflammatory cytokines [23].

#### Alternative pathway

Unlike the canonical and non-canonical pathways, which require K+ efflux and pyroptosis, the alternative pathway activates the NLRP3 inflammasome without these cellular events. This pathway is restricted to human and porcine monocytes and does not occur in murine cells. When human monocytes are exposed to LPS, the TLR4-TRIF-RIPK1-FADD-CASP8 signaling cascade enhances NLRP3 activation [37]. This signaling is distinct from classical NLRP3 inflammasome activation, which relies on K+ efflux and ASC speck formation. In addition to LPS, apolipoprotein C3 (ApoC3) has also been shown to activate NLRP3 inflammasomes through an alternative pathway. ApoC3 achieves this by forming a heterotrimer complex between TLR2, TLR4, and the SCIMP adaptor protein, triggering calcium entry, ROS production, and subsequent activation of caspase-8 [29]. The alternative pathway of NLRP3 inflammasome activation involves human monocytes through non-classical stimuli and mechanisms that bypass the typical priming and activation steps [38]. Human monocytes trigger caspase-1 to produce IL-1β without secondary stimuli when stimulated with LPS. This alternative inflammasome pathway does not cause ASC speck formation, ionic efflux of K^+^, or pyroptosis [39]. Extended LPS exposure without supplementary signals in murine dendritic cells results in NLRP3-mediated IL-1β processing and exudation in a P2X7R-independent way through TLR4-TRIF-RIPK1-FADD-CASP8 signaling pathway [23]. Unlike most PRRs, NLRP3 contributes to the post-transcriptional activation of the IL-1 class family, which serves as a final barrier before triggering an inflammatory reaction [25].

### Role of NLRP3 in PANoptosis

PANoptosis, an emerging inflammatory pathway, integrates three key forms of programmed cell death: apoptosis, pyroptosis, and necroptosis. This unique pathway regulates homeostasis and influences disease progression, particularly in tumorigenesis [40]. The concept of the PANoptosome, introduced by Christgen et al., highlights the direct interactions among PANoptotic molecules and the formation of this multiprotein complex, facilitating extensive crosstalk between the cell death pathways. PANoptosis operates through the coordination of the PANoptosome, enabling the integration of these cell death pathways, with the NLRP3 inflammasome playing a central role. Upon detecting Z-RNA, often produced during viral infections such as influenza, the NLRP3 inflammasome forms a complex with Z-DNA binding protein 1 (ZBP1), which recruits RIPK3 and caspase-8. This assembly activates the inflammasome, triggering cell death. Several distinct PANoptosome species have been identified, including the ZBP1-PANoptosome, AIM2-PANoptosome, RIPK1-PANoptosome, and NLRP12-PANoptosome, each shedding light on the diverse mechanisms driving immune responses and disease processes [41]. Aberrant expression and mutations of PANoptosis-related genes (PRGs), such as NLRP3, caspase-8, and TNFAIP3, have been identified across various cancers, with many PRGs acting as tumor risk factors. The increased expression of ADAR1-p150 in cancers restricts the interaction between ZBP1 and RIPK3, thereby suppressing ZBP1-mediated PANoptosis and facilitating tumor growth [42].

The therapeutic implications of targeting NLRP3 in PANoptosis are promising. NLRP3 inhibitors, such as MCC950, have demonstrated potential in reducing inflammation and enhancing anti-tumor immunity by repressing immune checkpoint expression and promoting apoptosis in cancer cells [43]. A study identified polyphyllin VI (PPVI) as a specific agonist that activates the ROS/NF-κB/NLRP3/GSDMD signaling pathway, effectively inhibiting NSCLC by preventing cancer cell proliferation [44]. Similarly, the chemotherapeutic agent cisplatin has been shown to exert anti-tumor effects in triple-negative breast cancer by inducing pyroptosis. This process occurs through the upregulation of the maternally expressed gene-3 and activation of the NLRP3/caspase-1/GSDMD signaling pathway, offering novel insights into the development of therapeutic strategies for the disease [45]. These findings highlight the significance of PANoptosis and its related genes as promising prognostic biomarkers and potential therapeutic targets in cancer; however, their role in IPF, remains underexplored, presenting an important avenue for future research.

### Role of NLRP3 in the Pathogenesis of IPF

IPF, a progressive and fatal form of ILD, is characterized by atypical tissue remodeling, excessive ECM deposition, and deteriorating lung function. Multiple cell types contribute to its pathophysiology, including fibroblasts, epithelial, immune, and endothelial cells. Key receptors like TGF-β, TLRs, and tenascin-C receptors are critical in disease progression [46]. The alveolar lining comprises AT1 and AT2 epithelial cells, facilitating gas exchange and producing surfactants [47]. Recurrent micro-injuries from dust, smoke, infections, or acid reflux can damage AECs, triggering persistent inflammation and EMT. This transition involves epithelial cells losing proteins like E-cadherin, which maintains cellular integrity, and adopting mesenchymal traits, enabling tissue migration [48]. The development of senescent fibroblasts, which resist apoptosis and exhibit a senescence-associated secretory phenotype (SASP), further amplifies disease progression by releasing cytokines and growth factors [49].

In IPF, fibroblastic foci proliferate during tissue repair and form MYFs, which embed and perpetuate fibrosis. TGF-β1, a central pro-fibrotic mediator, promotes EMT, fibroblast activation, and MYF differentiation. The PI3K/AKT signaling is a major pathway that regulates cellular functions like glucose metabolism, proliferation, and survival; its activation by AEC injury drives fibroblast proliferation and differentiation, leading to ECM deposition and α-SMA overexpression. Additionally, PI3K activation, mediated by Forkhead box (FOX) proteins, interacts with TGF-β1 in the fibrotic state to further amplify fibroblast activity, worsening IPF [46, 50].

**Fig. 2:**
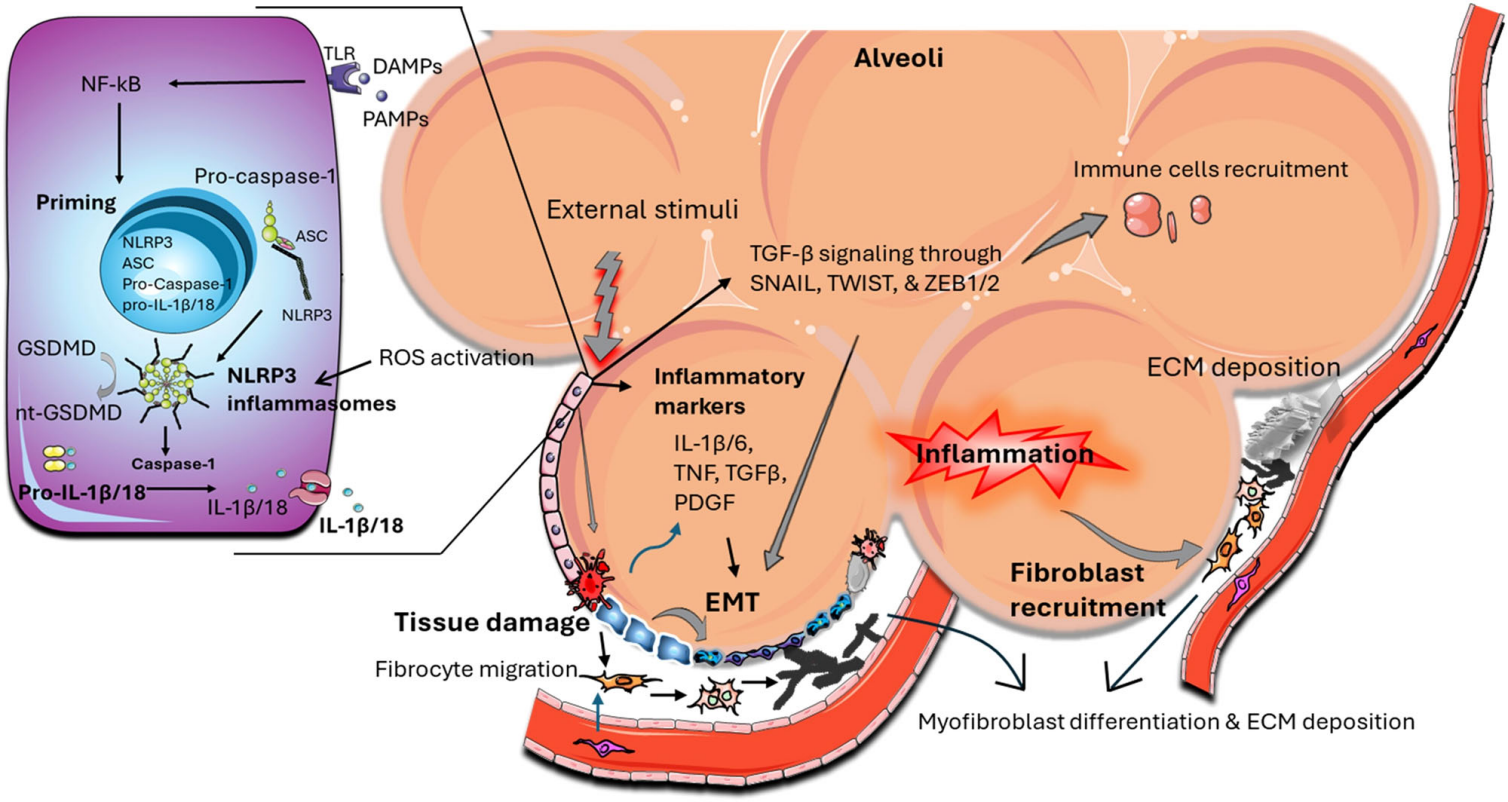
The role of nucleotide-binding domain, leucine-rich repeat, and pyrin domain-containing protein-3 (NLRP3) in idiopathic pulmonary fibrosis is to form the inflammasomes and promote the release of inflammatory markers, thereby causing myofibroblast differentiation and extracellular matrix deposition in the alveoli

Lungs are continually exposed to inhaled microbes, particles, and host-consequential danger signals. Thus, an active and regulated immune system is required to ensure protection against diseases. Therefore, several families of innate immune PPRs, such as TLRs, RLRs, and NLRs, which intervene in early signaling and regulation of inflammatory intermediaries, are present in the lungs [32]. Alveolar macrophages, alveolar epithelial cells, and endothelial cells express elevated levels of NLRP3 mRNA. As shown in Fig. 2, the triggering of the NLRP3 inflammasome is initiated by several stress signals, including DAMPs and PAMPs [23]. Upon activation, the inflammasome promotes the cleavage of pro-caspase-1 into its dynamic form, which in turn converts pro-IL-1β and pro-IL-18 into their active cytokine forms. These cytokines are central to the inflammatory response, contributing to the fibrotic process [28]. Mice deficient in NLRP3 or ASC does not have increased IL-1β, bronchoalveolar lavage fluid (BALF) inflammatory cells, or collagen deposition [25]. Therefore, it is conjectured that dysregulated instigation of NLRP3 may influence the advancement of chronic lung diseases such as IPF, asthma, and chronic obstructive pulmonary disease [51].

#### Inflammatory Cascade

In IPF, the activation of NLRP3 leads to the secretion of IL-1β and IL-18, which amplify the inflammatory response and recruit additional immune cells to the lung tissue [52]. IL-1β plays a particularly pivotal role by binding to the IL-1 receptor and activating downstream signaling pathways that include NF-κB and MAPK [53]. These pathways further stimulate the production of pro-fibrotic cytokines such as TGF-β (transforming growth factor-beta), a key driver of fibrosis [54].

#### Fibrogenic Response

TGF-β is a significant mediator of fibrogenesis in IPF. It induces the differentiation of fibroblasts into MYFs, which produce excessive amounts of ECM proteins, causing tissue scarring and lung stiffness [4]. The NLRP3 inflammasome facilitates this process by enhancing TGF-β signaling through various transcription factors such as SNAIL, TWIST, and ZEB1/2 [55]. These factors involve epithelial-mesenchymal transition (EMT), where epithelial cells acquire mesenchymal fibrogenic characteristics [55]. NLRP3 interacts with the ASC, a bipartite adapter protein that promotes the mobilization of pro-CASP1 to the inflammasome complex [25]. Active CASP1 cleaves the pro-cytokines (IL-1β and IL-18) into their biologically active forms [23] that promote collagen synthesis [54].

Several cell types are involved in the NLRP3-mediated fibrogenic response in IPF:

- ***Macrophages:*** They are primary sources of IL-18 and IL-1β following NLRP3 initiation. Alveolar macrophages, in particular, are critical in initiating and sustaining inflammation and fibrosis [56].
- ***Fibroblasts:*** Activated fibroblasts are the principal effector cells that produce collagen and other matrix components, leading to fibrosis. NLRP3 activation enhances fibroblast-to-myofibroblast transition [57].
- ***Epithelial Cells:*** These cells contribute to lung fibrosis through EMT, a process stimulated by NLRP3 activation and subsequent TGF-β signaling [58].

#### Therapeutic Implications

Inhibiting the NLRP3 inflammasomes offers a promising therapeutic approach for IPF. NLRP3, IL-1β, and caspase-1 inhibitors are being explored to mitigate the inflammatory and fibrogenic responses. Drug and phytochemical-based interventions aim to reduce fibrosis progression and preserve lung function by dampening the excessive inflammation and tissue remodeling associated with NLRP3 activation.

## Crude Extracts Acting on NLRP3 Inflammasome and Their Role in Fibrotic Diseases

Crude plant extracts are a hub of diverse phytochemicals, many of which have demonstrated the ability to modulate the NLRP3 inflammasome, thereby influencing the progression of fibrotic diseases and inflammatory conditions. For example, compounds isolated from crude extracts of *Andrographis paniculata* like, (8R–12S)-Isoandrographolide, directly downregulate inflammasome-related proteins such as NLRP3, ASC, and caspase-1. This inhibition has been linked to reduced pulmonary inflammation and fibrosis in rodent silicosis models [59]. Similarly, erianin, extracted from *Dendrobium chrysotoxumexerts*, shows inhibitory effects on the NLRP3 inflammasome by targeting the Walker A motif in the NACHT domain, thereby suppressing NLRP3 ATPase activity. This interaction has demonstrated therapeutic benefits in conditions such as peritonitis, gouty arthritis, and type 2 diabetes [60]. Agnuside, from the extract of *Vitex negundo*, reduces the expression of key inflammasome components, including NLRP3, caspase-1, and ASC, along with downstream inflammatory cytokines IL-1β and IL-18 and has been effective in alleviating synovitis and fibrosis in knee osteoarthritis models [61].

Trichospira verticillata extract inhibits NLRP3 activation by reducing its interaction with NEK7, which subsequently lowers ASC oligomerization and speck formation. This mechanism has shown efficacy in alleviating neutrophilic asthma symptoms in mice, indicating potential therapeutic applications for NLRP3-mediated diseases [62]. Likewise, Pomegranate Peel Extract targets the NLRP3/caspase-1/IL-1β signaling pathway, mitigating the expression of pyroptosis-related genes. This extract demonstrated cardioprotective properties, particularly in diabetic cardiomyopathy, where it reduces fibrosis and improves lipid metabolism [63]. *Alternanthera brasiliana* extract has been shown to alleviate liver injury and fibrosis induced by carbon tetrachloride (CCl_4_) in Balb/C mice by modulating NF-κB, MMPs, α-SMA, and collagen levels via the TGF-β/Smad axis [64]. Furthermore, shenlian extract, a TCM, has been found to inhibit NLRP3 inflammasome activity, reducing the secretion of inflammatory cytokines, and thereby providing protective effects against myocardial ischemic injury [65]. Likewise, *Radix angelica sinensis* and *Radix astragalus* extract have demonstrated the ability to inhibit radiation-induced pulmonary fibrosis by regulating the NLRP3/caspase-1/GSDMD signaling pathway, thereby reducing inflammation and fibrosis markers [66].

Herbal crude extracts contain multiple classes of phytochemicals that may contribute to their holistic therapeutic efficacy, particularly through NLRP3 inflammasome inhibition. However, limited research exists on flavonoid-rich extracts specifically evaluated for antifibrotic activity via this mechanism. Notably, a study has reported that the total flavonoid extract (TFE) from *Dracocephalum moldavica* mitigates pulmonary fibrosis by regulating pyroptosis pathways and autophagy, leading to decreased NLRP3 inflammasome activation [67]. Similarly, the TFE from *Psidium guajava* leaves has shown the potential to reduce pancreatic inflammation and fibrosis in chronic pancreatitis by preventing NLRP3 inflammasome activation [68].

## Flavonoids Acting on NLRP3 Inflammasomes and Their Role in IPF

As discussed in the previous section, crude drugs contain multiple bioactive components that can modulate the NLRP3 inflammasome. However, this section is focused specifically on individual flavonoids, their activity on NLRP3, and their potential therapeutic roles in treating idiopathic pulmonary fibrosis (IPF), as detailed in Table 1.

**Table 1:** Flavonoids-based therapeutic interventions in treating pulmonary fibrosis

Flavonoid	Target Pathways or Markers	Experimental Evidence	Mechanism of action	Ref.
Epicatechin	MAPK, NF-KB	BIPF rodents, LPS-induced ALI rodents.	Reduces mitochondrial dysfunction, oxidative stress, and inflammatory cytokines.	[74, 75]
	Nrf-2, NLRP3	Smoke-induced COPD rodents.	Reduces lung inflammation by inhibiting NLRP3 activation and Nrf-2 proteins.	[77]
Epigallocatechin-3-gallate	Surfactant protein A2 (SP-A2)	CHO-K1 cells.	Inhibits the aggregation of misfolded SP-A2 proteins.	[73]
	α-SMA	Silica-induced pulmonary fibrosis rodents.	Decreases hydroxyproline content, collagen accumulation and downregulates α-SMA.	[76]
Quercetin	SIRT-1, PKM2, NLRP3.	LPS-indued Lung Injury rodents.	Inhibits nuclear accumulation of PKM2, downregulates NLRPS, and activates SIRT-1 expression.	[85]
	SMA, Collagen-I and III, S1P.	HELF cells and BIPF rodents.	Inhibits SphK1/S1P signaling and decreases expression of markers. And collagen	[83]
	SASP, PTEN, PI3K, AKT.	RLE-6TN cells and BIPF rodents.	Inhibits cellular senescence, SASP cytokine secretion, and reputation PTEN/PI3K/AKT pathway.	[88]
Apigenin	Collagen I&III, Caveolin-1.	Paraquat-induced Pulmonary Fibrosis rodents.	Downregulates synthesis of Collagen (I and III), Inflammatory cytokines, and Caveolin-1.	[93]
	TNF- α, TGF- β.	BIPF rodents.	Suppresses Hydroxyproline content, myeloperoxidase activity, and levels of TNF- α and TGF-β	[95]
Galangin	α-SMA, E-cadherin, Vimentin.	BIPF rodents.	Decreases expression of Vimentin and α-SMA and reduces inflammatory cell activation (CD8^+^, CD4^+^, CD69^+^)	[106]
Morin	PPAR-γ, siPPAR-γ.	NIH-3T3 cells and BIPF rodents.	Inhibits transformation of fibroblasts into MYFs through PPARγ-glutaminolysis-DEPTOR signaling.	[114]
	NLRP3, NACHT.	LPS-induced ALI rodents.	Suppresses the protein levels of NLRP3 inflammasome, attenuates inflammatory cell count in the BALF, and improves SOD activity	[115]
Myricetin	HSP90β, TGF-β.	A549 cells, MLE12 cells, Mlg cells, HLL1 cells, NIH-3T3 cells, and BIPF rodents.	Attenuates BIPF and inhibits TGF-β1-activated morphogenesis of lung fibroblasts and EMT by targeting HSP90β. Acts via suppressing TGF-β1/Smad signaling.	[123]
Dihydromyricetin	p-STAT3, TGF-β1, GLUT1.	Lung Tissues from IPF patients, and BIPF rodents.	Reduces the proliferation, uncharacteristic migration, and respiratory functions of MYFs induced by TGF-β1. Acts via the STAT3/p-STAT3/GLUT1 signaling pathway.	[124]
Biochanin-A	TIMP-I, TIMP-III, LOXL-2, α-SMA, TGF-β, Smad 2 and 3.	LL29 cells, NHLF cells, DHLF cells, and BIPF rodents.	Reduces the expression of TGF-β modulated fibrotic genes, inflammatory markers in BALF, and collagen deposition. Attenuates TGF-β/Smad2/3 phosphorylation.	[129]
Baicalein	α-SMA, Hydroxyproline, TGF-β1, TNF-α.	Rat primary fibroblast cells, BIPF rodents.	Increases glutathione peroxidase, superoxide dismutase, and glutathione levels and reduces EMT. Acts via inhibiting PI3K/AKT and CaMKII signaling pathways.	[138]
	α-SMA, Collagen1A1, STAT3, Smurf2.	MRC-5 cells	Reduces the levels of α-SMA mRNA and protein expression and the formation of collagen type I and fibronectin. Diminishes the expression of miR-21 transcriptor STAT3.	[139]
	SIRT3, MCP-1, PAI-1, TNF-α, MMP-10 and 12, p53, 21 and 16.	BIPF rodents	Decreases levels of collagen deposition, hydroxyproline, collagen I, MCP-1, PAI-1, and TNF-α. Downregulates SIRT3 expression in lung tissues by inhibiting the TGF-β1/Smad signaling pathway.	[140]
	NLRP3, Caspase-1, GSDMD	LPS-induced lung injury rodent.	Inhibits the NLRP3/Caspase-1/GSDMD pathway, reducing pyroptosis and protecting lung LPS-induced tissue injury.	[143]
Naringenin	Hydroxyproline, Collagen, PINK1, ATF3	BIPF rodents	Maintains mitochondrial homeostasis by inhibiting PINK1 transcription, reducing ER stress, mitophagy-related genes, and ATF3 expression.	[146]
	IL-8, TNF-α, Nrf2, NQO1, SOD	BEAS-2B cells	Stabilizes the antioxidant system by modulating the Nrf2 pathway and its downstream regulatory genes like HO-1 and NQO1.	[147]
Luteolin	TGF-β1, α-SMA, Collagen, E-cadherin, Fibronectin, Vimentin, Smad3, TNF-α, IL-6,	A549 cells, Human fibroblast cells, and BIPF rodents	In cells, it suppresses α-SMA, type I collagen, and vimentin expression. Blocks TGF-β1-mediated EMT and downregulates E-cadherin. In rodents, it reduces neutrophil infiltration, as well as the levels of TNF-α and IL-6 in bronchoalveolar lavage fluid. Also, alleviates collagen deposition, reduces TGF-β1 expression, and suppresses lung fibrosis.	[156]
	E-cadherin, TGF-β1, PI3K-AKT, IκBα, NF-κB, EMT	A549 cells	Inhibits the activation of the PI3K-AKT-IκBα-NF-κB-Snail pathway, which is typically involved in promoting EMT and the loss of E-cadherin.	[157]
Kaempferol	TGF-β1, E-cadherin, α-SMA, N-cadherin, PAR-1, EMT	BEAS-2B cells and OVA-sensitized rodents.	In cells, it inhibits TGF-β-induced EMT in BEAS-2B cells. Reverses the loss of E-cadherin expression and the induction of α-SMA and N-cadherin. In rodents, it alleviates airway fibrosis by reducing collagen deposition, and specific inhibition of TGF-*β* and PAR-1 which restores E-cadherin and reduces α-SMA.	[165]
Pinocembrin	CD8^+^ and CD4^+^ T cells, Hydroxyproline	BIPF Sheep	Remodels lung tissue by decreasing connective tissue buildup, inflammation, and immuno-positive T- cells in the lung parenchyma.	[173]
	TLR4, NF-κB, NLRP3	LPS and BIPF rodents.	Inhibits the TLR4-NF-κB signaling pathway, thereby preventing activation of pro-inflammatory cytokines. Suppresses the NLRP3 inflammasome, reducing the activation of caspase-1 and subsequent release of inflammatory cytokines.	[174]
Chrysin	TGF-β1, TXNIP, Hydroxyproline, HIF-1α.	BIPF rodents.	Reduces hydroxyproline content, TGF-β1 expression, inflammatory cell infiltration, and LDH activity. Enhances antioxidant defense (SOD, GSH) and decreases nitric oxide, TXNIP, and HIF1α, indicating reduced tissue hypoxia.	[178]
	GRP78, p-IRE1α, TXNIP, NLRP3, caspase-1	LPS-induced ALI rodents.	Reduces inflammation in ALI by inhibiting the IRE1α/TXNIP/NLRP3 inflammasome pathway. Reduces pro-inflammatory cytokine release and MPO activity while improving redox homeostasis.	[181]
Diosmin	T-cell receptors, IL-2, IL-17, NF-κB, p-IκB-α.	LPS-induced ALI rodents.	Reduces oxidative stress, inhibits pro-inflammatory cytokines (IL-2, IL-17, TNF-α), downregulates the expression of IL-6, IL-1β, and TNF-α, mitigates the activation of NF-κB signaling.	[187]
	GSH, NF-κB, MAPK, TNF-α.	Paraquat (PQ)-induced pulmonary fibrosis rodents.	Reduces oxidative stress, inflammation, and fibrosis by enhancing antioxidant levels (GSH, catalase), and inhibits NF-κB, MAPK, and TNF-α activation.	[185]
Nobiletin	NLRP3, PI3K, AKT, mTOR, NLRP3, α-SMA, TGF-β1.	Amiodarone-induced pulmonary fibrosis rodents.	Reduces ROS production, activating the Nrf2/HO-1 pathway. Inhibits NLRP3 inflammasome activation, reduces IL-1β release, suppresses the PI3K/AKT/mTOR fibrotic pathway, and lowers α-SMA expression and collagen deposition.	[192]

### Catechin, Epicatechin, Epicatechin-3-gallate, and Epigallocatechin-3-gallate

Catechins, including Catechin (CA), Epicatechin (EC), Epicatechin-3-gallate (EPG), and Epigallocatechin-3-gallate (EGCG), are flavanols widely found in foods such as wine, tea, black grapes, strawberries, and apricots, with green tea being particularly rich in EGCG [69]. These compounds exhibit potent antioxidant and anti-inflammatory properties, enhancing muscle performance, improving cardiovascular and cerebrovascular health, diabetes mellitus (DM) prevention, and neuroprotection [70].

In studies involving human lung fibroblast cells exposed to amiodarone, both EC and CA effectively modulated mitochondrial dysfunction and oxidative damage, highlighting their protective effects against drug-induced toxicity [71]. EPG has been demonstrated to inhibit tumor angiogenesis, with studies on human melanoma cells showing that both EPG and EGCG induce apoptosis and inhibit cell proliferation, with EPG exhibiting more potent inhibitory effects [72]. Meanwhile, EGCG has demonstrated the ability to inhibit the aggregation of misfolded surfactant protein A2 (SP-A2), proposing a prospective role in mitigating the pathogenesis of lung fibrosis associated with protein aggregation [73].

EC has shown protective impacts against BIPF by reducing mitochondrial dysfunction and oxidative stress [74]. EC alleviates acute lung injury in BALB/c mice (ALI) induced by LPS and inhibits inflammatory cytokines and critical signaling pathways, such as MAPK and NF-κB [75]. EGCG has shown promising effects in treating irradiation-induced and silica-induced pulmonary fibrosis in Sprag Dawley rat models [76]. In smoke-induced COPD rat models, EC enhances the nuclear localization of the Nrf2 protein, inhibits NLRP3 inflammasome activation, and reduces lung inflammation [77]. EGCG supplementation has decreased levels of pro-inflammatory cytokines (IL-1, IL-1β, and TNF-α) in seawater aspiration-induced ALI [78]. EGCG inhibits LPS/AβO-induced neuroinflammation by targeting the ROS/TXNIP/NLRP3 pathway, thus attenuating NLRP3 inflammasome activation by suppressing caspase-1 activation, IL-1β secretion, and NLRP3-mediated ASC speckle formation [79]. Additionally, in vitro and preclinical studies have shown that EGCG attenuates Hla-induced NLRP3 inflammasome activation, suggesting its effectiveness in reducing inflammatory responses mediated by bacterial toxins [80].

Beyond pulmonary health, EGCG has demonstrated broader health benefits, including improving glucose forbearance in high-fat diet-induced type-2 DM models and suppressing vascular inflammation in human endothelial cells [81]. Catechins, particularly EGCG, EC, and EPG, represent a promising class of compounds with multifaceted roles in mitigating oxidative stress, inflammation, and fibrosis across various disease models via the NLPR3 inflammasome pathway.

### Quercetin

Quercetin is a flavanol and abundantly found polyphenol in the human diet [82]. As mentioned in Table 2, it has been reported to have various pharmacological activities. Studies on mouse models of IPF have shown that quercetin treatment reduces fibrosis markers such as α-SMA, Collagen I, and Collagen III via sphingosine-1-phosphate (S1P) signaling while increasing E-cadherin and LC3II/LC3I levels, indicating enhanced autophagy [83]. Quercetin in Jie-Geng-Tang formulation [84] has been demonstrated to reduce inflammatory cytokine concentrations, diminish fibrosis, and attenuate collagen deposition, highlighting their potential in managing EMT deposition.

**Table 2:** Overview of Flavonoids with Anticancer, Antifibrotic, Anti-inflammatory, and Antioxidant Activities; Structural Classification, Examples, and Sources

Class	Structural features	Examples	Sources	Biological Activities	Chemical Structure*
Flavones	Flavones are a class of flavonoids characterized by a 15-carbon skeleton comprising two benzene rings (A and B) connected by a heterocyclic pyran ring (C). They feature a carbonyl group at C4 in the C ring and a double bond between C2 and C3. The attachment of the B ring is at the 2-position. Flavones often have hydroxyl groups at positions 5, 7, 3′, 4′, and 5′ but do not have a hydroxyl group at position 3. In nature, these hydroxyl groups can be encountered as methyl ethers or acetyl esters, and it is typical for sugars such as galactose, arabinose, rhamnose, or glucose to be attached to these hydroxyl groups at positions 3 or 7) [202].	Apigenin	Parsley, chamomile, celery, vine-spinach, artichokes, and oregano [203].	Antioxidant [204] Anti-inflammatory & Anticancer [205] Antifibrotic [206]	
		Luteolin	Celery, Parsley, Broccoli, Onion Leaves, Carrots, Peppers, Cabbages, Apple Skins, and Chrysanthemum Flowers.	Antioxidant [207] Anti-inflammatory [208] Anticancer [209] Antifibrotic [210]	
		Targretin	Citrus fruit peel.	Antioxidant & Anti-inflammatory [211] Anticancer [212] Anti-asthmatic [213]	
		Nobiletin	Citrus fruit peel.	Antitumor [214] Antifibrotic [192] Anti-inflammatory [211]	
		Diosmetin	Citrus Fruits, Rosemary leaves, and olives.	Antioxidant [215] Anti-inflammatory [216] Antitumor [217] Antifibrotic [218]	
		Baicalein	*Scutellaria baicalensis*	Antioxidant [219] Anti-inflammatory [220] Antifibrotic [221]	
		Chrysin	Honey	Antioxidant & Anti-inflammatory [222] Antitumor [223] Antifibrotic [224]	
Flavonols	Flavonols, or 3-hydroxyflavones, are a distinct subgroup of flavonoids characterized by specific structural features. These compounds possess hydroxyl groups at the 5 and 7 positions on the A ring, while the B ring is connected to the A ring via a three-carbon chain. A defining characteristic of flavonols is the presence of a hydroxyl group at the 3-position on the C ring, distinguishing them from other flavonoid subclasses. This hydroxyl group may undergo glycosylation, contributing to the diversity of flavonols. Like flavones, flavonols exhibit various methylation and hydroxylation patterns, enhancing their structural diversity [203].	Kaempferol	Kale, beans, tea, broccoli, apples, and strawberries.	Antioxidant [225] Anti-inflammatory [226] Anticancer [227]	
		Rutin	Buckwheat, citrus fruits, apple, berries, and green tea.	Antioxidant [228] Anti-Cancer [229] Antifibrotic [230] Anti-inflammatory & Antitumor [231]	
		Myricetin	Black currants, cranberries, and goji berries.	Antioxidant [232] Anti-Cancer [233] Anti-Inflammatory [233] Antifibrotic [123]	
		Quercetin	Onions, apples, grapes, berries, broccoli, citrus fruits, cherries, tea, and red wine.	Antioxidant [234] Anti-Inflammatory [235] Antiallergic [236]	
		Fisetin	Strawberries, apples, persimmons, onions, and cucumbers.	Antioxidant [237] Anti-Cancer Anti-Inflammatory [237] Antifibrotic [238]	
		Galangin	*Alpinia officinarum*	Antioxidant [239] Anti-inflammatory Antitumor [240, 241] Antifibrotic [106]	
Flavanones	Flavanones, also known as dihydroflavones, differ structurally by having a saturated C ring, meaning the double bond between positions 2 and 3 is fully reduced. They share the same 15-carbon skeleton as other flavonoids, with two aromatic rings (A and B) and a heterocyclic C ring. A characteristic carbonyl group is present at the C4 position of the C ring in flavanones. Additionally, flavanones can be hydroxylated at various positions, most commonly at the 5 and 7 positions on the A ring, with these hydroxyl groups often undergoing glycosylation or methylation. This hydrogenation and varied hydroxylation confer distinct biological properties to flavanones [242].	Hesperetin	Citrus fruits.	Antioxidant, Antibacterial & Anti-inflammatory [243] Anticancer [244] Antifibrotic [245]	
		Hesperidin	Citrus fruits.	Antioxidant, Antibacterial, Anti-inflammatory [243] Antifibrotic [246]	
		Naringenin	Citrus fruits, tomatoes, and grapefruits.	Antioxidant & Anti-inflammatory [222] Anticancer [247] Antifibrotic [147]	
		Pinocembrin	Damiana, Honey, propolis, and fingerroot.	Antioxidant [248] Anti-inflammatory and Anticancer [249] Antifibrotic [173]	
Flavononols	Flavanonols, or dihydroflavonols, are a subdivision of flavonoids represented by a C15 (C6-C3-C6) structure comprising two aromatic rings (A and B) and a heterocyclic C ring. A distinctive feature of flavanonols is the presence of a hydroxyl group at the 3-position of the C ring. Like flavanones, the C2-C3 bond in flavanonols is saturated, differentiating them from flavones. Flavanonols also possess two chiral centers at the 2nd and 3rd positions of the C ring, adding to their structural diversity, which includes compounds like catechin and epicatechin. The varied hydroxylation and methylation patterns in flavanonols enhance their solubility, stability, and biological activities, particularly their antioxidant properties.	Taxifolin	Milk thistle and onions.	Antioxidant [250] Anti-inflammatory [251] Antitumor [252] Antifibrotic [253]	
		Genistein	Soybeans and chickpeas.	Antioxidant [254] Anti-inflammatory [255] Anticancer [256] Antifibrotic [255, 257]	
Isoflavones	The B-ring is attached to the third position of the C-ring. They are also called phytoestrogens because they are structurally similar to estrogens such as estradiol.	Daidzein	Soybeans and chickpeas.	Antioxidant [254] Anti-inflammatory [258] Anticancer [259]	
		Biochanin A	Red clover, alfalfa, and peanuts.	Antioxidant [260] Anticancer [261] Antifibrotic [129]	
Neoflavonoids	B ring attached to position 4 of the C ring.	Neohespiridin	Citrus fruits.	Antioxidant [262] Anti-inflammatory [263] Anticancer [264] Antifibrotic [265]	
Flavanol	Flavanols, or flavan-3-ols, are characterized by the hydroxyl group bound to position 3 of the C ring. Their lack of a double bond between positions 2 and 3 differs from many other flavonoids [242].	Catechin	Tea (especially green tea), cocoa, apples and berries.	Antioxidant [266] Anti-inflammatory [267] Anticancer [268] Antifibrotic [269]	
		Epicatechin	Tea (especially green tea), cocoa, apples and berries.	Antioxidant [270] Anti-inflammatory [271] Antifibrotic [74]	
		Morin	*Maclura pomifera*, *Maclura tinctoria*, and leaves of *Psidium guajava*.	Antioxidant [272] Anti-inflammatory [273] Antifibrotic [272]	
Anthocyanidins	Anthocyanidins are flavylium cations and generally exist as chloride salts. These are the only group of flavonoids that give plants their color. Glycosylated anthocyanidins are called anthocyanins. The sugar moieties are mostly attached to the 3-position of the C ring and are often conjugated to phenolic acids such as ferulic acid.	Cyanidin	Berries, grapes, red cabbage, and plums.	Antioxidant [274] Anti-inflammatory [275] Anticancer [276]	
		Delphinidin	Berries, grapes, and pomegranates.	Antioxidant [277] Anti-inflammatory [275] Anticancer [278]	
Chalcones	Chalcone and dihydrochalcone are open-structured flavonoids. They are classified as flavonoids because they share a similar synthetic pathway.	Flavokawain A	Kava plant.	Antioxidant [279] Anti-inflammatory [280] Antifibrotic [279]	
		Cardamonin	Kava plant and various species of the *Alpinia* genus.	Antioxidant [281] Anti-inflammatory [282] Anticancer [283]	

*All the chemical structures were downloaded from PubChem (nih.gov)

In an endotoxin-induced ALI rat model, quercetin downregulated NLRP3 inflammasome initiation by preventing the nuclear deposition of PKM2 and upregulating SIRT1 expression [85]. In mice, quercetin restores collagen-induced arthritis by inhibiting the TLR-4/NLRP3/p-65 pathway and TH17/Treg balance by hindering NLRP3 signaling [86]. In STZ-induced diabetic rats, quercetin has been shown to have a protective effect on the lungs by restoring the balance of oxidant/antioxidant markers such as MDA, SOD, and GSH. Moreover, it downregulates the expression of pulmonary protein markers such as NLRP3, Cas1, GSDMD, and IL-1β mRNA expression in pulmonary tissue [87].

In BLM-induced pulmonary fibrosis rats, quercetin has been found to inhibit macrophage senescence, reduce fibrosis, and regulate gut microbiota by boosting the abundance of Akkermansia via PTEN/PI3K/AKT signaling pathway [88]. These results highlight the possible mechanisms of quercetin through the SIRT1/NLRP3 inflammasome pathway. However, further exploration is needed to strengthen the correlation.

### Apigenin

Apigenin is a naturally found flavonoid in various vegetables, fruits, and herbs, such as celery, parsley, chamomile, onions, and maize [89]. Chemically known as 4′,5,7-trihydroxyflavone, it belongs to the flavone class, appears as a yellow crystalline powder, and possesses numerous pharmacological activities as listed in Table 2 [89]. Apigenin targets biological processes related to inflammation, aging, and cancer through pathways such as FOXO, mTOR, NF-κB, and PI3K-Akt [90]. Studies have reported that apigenin treatment can prevent the expression of fibrosis-related cytokines like TGF-β1, TNF-α, and matrix metalloproteinase-9 while increasing lung glutathione and PPARγ expression [91].

Apigenin has been found to reduce the production of type-I interferons in response to STING pathway agonists, thereby mitigating pathological pulmonary inflammation and lung edema in ALI [92]. In mice, apigenin shields against paraquat-induced lung fibrosis by down-regulating type-I and type-III collagen content, inflammatory cytokine levels, and Caveolin-1 protein expression [93]. Apigenin also inhibits renal fibroblast proliferation, differentiation, and function by activating AMPK and reducing ERK1/2 phosphorylation, suggesting its potential as a therapeutic agent for renal fibrosis [94]. Furthermore, it prevents BIPF in rats by suppressing increased hydroxyproline content, myeloperoxidase activity, TNF-α, and TGF-β levels and inhibiting excessive collagen deposition [95].

Apigenin has demonstrated potential in alleviating non-alcoholic fatty liver disease (NAFLD) and atherosclerosis by inhibiting NLRP3 inflammasome activation in mice with high-fat diets [96]. Studies in rats and mice indicate that apigenin treatment reverses depressive-like behaviors induced by chronic mild stress and LPS, ameliorating depressive behavior by hindering IL-1β synthesis and NLRP3 inflammasome assembly in the brain [97]. Inhalable formulations of apigenin loaded in bovine serum albumin (BSA) nanoparticles show promise for efficient pulmonary transport, exhibiting antioxidant activity and potential as a novel delivery system against lung injury [98]. Apigenin has been found to reduce oxidative stress markers in paraquat [99] and irradiation [100] lung injury models, thereby increasing the levels of catalase, glutathione, and superoxide dismutase while decreasing concentrations of malondialdehyde and myeloperoxidase. However, detailed human pharmacokinetic studies must determine apigenin’s initial safety and tolerability before conducting large-scale clinical trials.

### Galangin

Galangin (3,5,7-trihydroxyflavone) is a flavonoid found in various herbs like *Crocus sativus*, *Alpinia officinarum*, and *Plantago major* [101]. It is a crystalline powder with a yellow tint, has poor water solubility, and exhibits numerous bioactivities, as shown in Table 2. In rats, galangin undergoes metabolism (Phase I and II), producing metabolites like chrysin, kaempferol, and apigenin, which also have significant bioactivities [102]. Long-term administration can alter CYP450 enzyme activity, potentially improving oral drug bioavailability [103].

Liver fibrosis, induced by factors like viruses, alcohol, and drug abuse, can be suppressed by galangin, which scavenges oxidative free radicals, reduces the peroxidation of lipids, and prevents the activation and production of hepatic stellate cells (HSCs) [104]. Yuoung et al. reported that galangin considerably converses hepatic fibrosis and causes HSCs apoptosis by interrupting the PI3K/Akt, Bax/Bcl-2, and Wnt pathways, essential in disease management [105]. Similarly, galangin significantly alleviates BIPF in mice by preventing the expression of vimentin and α-SMA while increasing E-cadherin expression. It also reduces inflammatory cell activation, such as CD8^+^, CD4^+^, CD69^+^, T cells, and dendritic cells, thereby decreasing inflammation in pulmonary tissue [106]. Pretreatment with galangin in ISO-induced rats lowers heart rate, lipid peroxidation, and blood pressure. It inhibits the upregulation of IL-1β, TNF-α, NF-κB, and other related proteins, reducing inflammation, oxidative stress, and cardiac fibrosis [107].

Lu et al. reported that in NRK-52E cells treated with uric acid, galangin diminished IL-1β, PGE2, TNF-α, NO, iNOS, IL-18, and PTGS2 levels. This effect was achieved by curbing the activation of the NF-κB, PI3K/AKT, and NLRP3 pathways, thereby alleviating the renal inflammatory response [108]. These findings suggest that galangin can relieve fibrosis in multiple organs by inhibiting fibrotic genes and reducing oxidative stress. However, limited studies have shown galangin’s ability to modulate the NLRP3 inflammasome and its anti-inflammatory properties for treating lung fibrosis.

### Morin

Morin is a natural pyran ring flavonoid found in Moraceae plants, like Moringa, often called the “Miracle Tree” [109], and has versatile pharmacological activities, as shown in Table 2. In MDA-MB-231 breast cancer cells, morin suppresses the EGFR/STAT3 pathway due to the suppression of FOXM1 and chemosensitization of tumor cells [110]. Additionally, morin inhibits colorectal cell proliferation and tumors in vivo, suppresses TNF-α-induced p65-NFκB expression, and reduces the production of IL-8 and IL-6 [111]. Morin influences pathways such as MAPK and NF-κB, causing mitochondrial-mediated apoptosis and autophagy, cell cycle arrest, and apoptosis [112]. In combination with cisplatin, morin reduces the expression of galectin-3 in ovarian tumor cells at the mRNA and protein levels [113].

Mechanistic studies have revealed that morin exerts its antifibrotic effects by triggering peroxisome proliferator-activated receptor-gamma (PPAR-γ) and restricting glutaminolysis, thereby inhibiting the transformation of fibroblasts into MYFs, which is crucial for developing fibrosis [114]. Additionally, Zhang Tianzhu et al. have reported that morin suppresses the NLRP3 inflammasome, attenuates inflammatory cell count in the BALF, and improves SOD activity, thereby inhibiting inflammatory response contributing to fibrosis progression in LPS-induced ALI [115]. By targeting these pathways, morin effectively reduces fibrosis at the cellular level. These findings highlight morin’s potential as a therapeutic agent for managing and preventing fibrotic diseases. However, further clinical studies need to be performed.

### Myricetin

Myricetin, a natural flavonol found in fruits, vegetables, wine, tea, and medicinal plants, exhibits various pharmacological properties. Myricetin prevents tumor development by promoting apoptosis through intrinsic and extrinsic pathways by networking with oncoproteins such as Akt, MEK1, Fyn, and JAK1-STAT3, thereby attenuating the neoplastic alteration of tumor cells [116]. It has been shown to reduce inflammatory responses by attenuating the upregulation of COX2 induced by UV exposure, activation of TGFβ/Smad, and repression of MAPK/AP-1 [117].

In A549 cells, myricetin deactivates TNF-α-induced inflammation by triggering the NF-κB pathway through SIRT1 activation [118]. Furthermore, myricetin modulates MAPK-related functions and signaling pathways, demonstrating anti-NSCLC effects directly binding to MKK3 and affecting the downstream p38-MAPK signaling pathway [119]. It inhibits the production and colonization of H1975 cells by affecting MMP1, MMP3, MMP9, and PIK3R1 expression levels, thereby controlling the multiple pathways [120].

Myricetin has been found to prevent NLRP3 inflammasome activation by ROS-independent ubiquitination of NLRP3 and reducing ROS-dependent ubiquitination of ASC [121]. This disruption in the connection linking ASC and NLRP3 inhibits ASC oligomerization. This effect was established in vivo using mice models of sepsis and peritonitis, suggesting the beneficial value of myricetin in targeting NLRP3-driven inflammatory diseases [121]. Myricetin has been shown to reduce NLRP3 inflammasome activation in STZ-induced diabetic rat models [122]. Myricetin inhibits TGF-β1-activated morphogenesis of lung fibroblasts and EMT by targeting HSP90β, indicating its potential as a therapeutic agent for pulmonary fibrosis [123]. Similarly, dihydromyricetin has demonstrated the ability to normalize the differentiation of fibroblasts into MYFs and to reduce the proliferation, uncharacteristic migration, and respiratory functions of MYFs induced by TGF-β1 or those derived from patients with IPF [124]. Additionally, it has shown efficacy in alleviating BIPF in mice models via the inhibition of the STAT3/p-STAT3/GLUT1 signaling pathway [124].

### Biochanin-A

Biochanin-A is an isoflavone phytoestrogen and mimics the activity of estrogens. It is derived from various plants, including chickpeas, red clover, and soybean [125]. Biochanin-A modulates SIRT1 expression and interacts with PPARγ receptors to exert anti-diabetic effects [126]. Moreover, it positively impacts the rumen microbial community, promoting rumen fermentation and preventing rumen acidosis [127]. Biochanin-A influences cellular signaling pathways by regulating the PI3K/Akt cascade, MAPK signaling cascade, and NF-κB [126]. These pathways are critical in oxidative stress, neuroinflammation, and apoptosis linked with neurodegenerative diseases. Biochanin-A has also been reported to modulate the MAPK/NF-κB axis, which results in reduced expression of inflammatory cytokines and decreased myeloperoxidase activity [128].

Research on LL29 (IPF lung fibroblasts), NHLF (normal lung fibroblasts), and DHLF (diseased lung fibroblasts) cells showed that biochanin-A increased Smad7 expression and decreased Smad2 mRNA expression, indicating inactivation of the TGF-β/Smad signaling pathway [129]. Biochanin-A reversed the increased lung index in BIPF models, suggesting its potential to prevent IPF progression [129]. Biochanin-A was also studied for its antifibrotic effects in a rat liver model where fibrosis was induced with chloroform. The results showed reduced fibrotic lesions and α-SMA levels, improved blood flow and antioxidant properties, and reduced TNF-α and NO levels [130]. Biochanin-A exhibits cardioprotective effects by inhibiting the TLR4/NF-κB/NLRP3 inflammatory pathway, thereby reducing myocardial injury. It decreases the injury area, lowers cardiac enzymes, and reduces inflammatory cytokines, providing significant protection against myocardial infarction in rat models [131]. Although specific molecular mechanisms detailing the interaction between biochanin-A and NLRP3 are not well established in IPF, the compound’s cardiac and neuroprotective effects have been associated with triggering the PI3K/Akt cascade and preventing the MAPK signaling cascade, indicating potential molecular crosstalk that could regulate the NLRP3 inflammasome.

### Baicalein

Baicalein, chemically referred to as 5,6,7-trihydroxy-2-phenyl-4H-chromen-4-one, is a prominent flavonoid obtained from *Scutellaria baicalensi*s, one of the most abundant plant extracts used in TCMs [132]. Baicalein attenuates cardiac hypertrophy and fibrosis induced by pressure overload, potentially suppressing MEK-ERK1/2 signaling [133]. It activates the Nrf2 signaling pathway in Hct116 cells (colon carcinoma), with its derivatives showing comparable redox-active potential [134]. In NSCLC, baicalein down-regulates the expression of CyclinD1, CDK1, Twist1, and Vimentin via the Notch1and hes-1 signaling to indicate its prospective as a molecular target for therapy in H1299 and A549 [135]. By regulating the ERK signaling pathway, Baicalein prevents cell development, metastasis, and EMT progress in osteosarcoma MG-63 cells [136].

Baicalein has been found to attenuate peritoneal fibrosis by regulating cell proliferation, inflammatory responses, and the AGE-RAGE signaling pathway [137]. In a BLM-induced acute lung injury study, Baicalein significantly reduced pulmonary inflammation and early-stage EMT via the PI3K/AKT signaling pathway [138]. Baicalein also reduced hydroxyproline content and α-SMA levels, lowered the intensity of alveolitis and lung fibrosis, and increased total antioxidant capacity [138]. Additionally, baicalein decreases TGF-β1-induced human pulmonary fibroblast differentiation by preventing the expression of miR-21, a key regulator of fibroblast-to-myofibroblast differentiation [139]. Baicalein mitigates lung fibrosis by regulating the TGF-β1/Smad signaling pathway and preserving lung SIRT3 expression, thus suppressing fibroblast senescence and activating TGF-β1/Smad signaling [140]. A meta-analysis revealed that baicalein exerts protective effects against the progression of lung injury at a dose of 10 mg/kg to 200 mg/kg through anti-inflammatory and anti-apoptotic pathways by modulating NF-κB–p65, Akt, and Bcl-2–Bax–caspase-3 signaling pathways [141]. Moreover, baicalein has demonstrated therapeutic uses in treating several pulmonary diseases, such as COPD, asthma, pulmonary infections, ALI, acute respiratory distress syndrome, and lung cancer [142].

Baicalein has been demonstrated to inhibit the NLRP3/Caspase-1/GSDMD pyroptosis pathway, protecting against LPS-induced lung tissue injury. By targeting the NLRP3/Caspase-1/GSDMD pathway, baicalein reduces pyroptosis, thereby preserving lung tissue integrity and function [143]. Additionally, baicalein, found in Jinzhen Oral Liquid, inhibits the TLR4-MyD88-NF-κB/NLRP3 and MAPK signaling pathways in lung tissue injury induced using LPS [144]. This dual inhibition makes baicalein a potential candidate that reduces bronchial epithelial layer damage, alveolar damage, and pulmonary edema in acute lung injury via the NLRP3 inflammasome pathway.

### Naringenin

Naringenin, a characteristic dihydro-flavonoid found in citrus fruits, tomatoes, and medicinal plants [145], exhibits potent biological activities, as shown in Table 2. In a study using a BIPF mouse model, naringenin maintained mitochondrial homeostasis by impeding the transcription of PTEN-induced kinase 1 (PINK1), thereby inhibiting expression of endoplasmic reticulum stress, mitophagy-related genes, and activated transcription factor (ATF)-3 [146]. Naringenin’s protective effects on lung health are also attributed to its ability to stabilize the antioxidant system by altering the Nrf2 pathway and its downstream regulatory genes in BEAS-2B cells when treated with cigarette smoke extract [147].

Moreover, naringenin has shown promising results in protection against mild acute pancreatitis (MAP) induced by caerulein and severe acute pancreatitis (SAP) induced by L-arginine in mouse models [148, 149]. In these models, naringenin significantly reduced the recruitment of inflammatory cytokines such asTNFα, IL-6, and IL-1β and enhanced antioxidant enzymes such as glutathione reductase, glutathione peroxidase, glutathione S-transferase, and non-protein sulfhydryls [148, 149]. It also enhanced the expression of Nrf2/HO-1 in pancreatic tissue and impaired the activation of the NLRP3 inflammasome and IL-1β production [148, 149]. One of the significant complications of SAP is ALI [148]. Naringenin inhibits the NLRP3/NF-κB pathway in kupffer cells and hepatocytes, reducing lipid deposition and inflammation in NAFLD. These effects were dependent on NLRP3 expression, as re-expression restored its anti-lipid deposition impact in NLRP3-/- hepatocytes [150]. Furthermore, naringenin has been shown to reduce the expression of NLRP3, caspase-1, IL-1β, and IL-18 in high-glucose-induced inflammatory conditions, highlighting its potential as a novel treatment [151].

### Luteolin

Luteolin, a polyphenolic (benzo-γ-pyrone) flavonoid, is found in various fruits, vegetables, flowers, and herbs [152]. Traditionally used in Asian medicine for diseases like arthritis, rheumatism, hypertension, neurodegenerative disorders, and infections, luteolin induces apoptosis in cancer cells, inhibits cell proliferation, and regulates glycolipid metabolism [152]. Its mechanisms of action involve pathways such as NF-κB, toll-like receptor, MAPK, Wnt/β-catenin, PI3K/Akt, AMPK/mTOR, and Nrf-2, and it regulates cholesterol metabolism and glucose intolerance via the liver X receptor (LXR) α signaling pathway [153–155].

Studies have shown that luteolin in the pulmonary system can suppress neutrophil infiltration, reduce TNF-α and IL-6 levels, decrease collagen deposition, and lower TGF-β1 expression in bleomycin-instilled mice [156]. It has been shown to inhibit TGF-β1-induced type I collagen, α-SMA, and vimentin expression in mouse lung fibroblasts, maintaining epithelial morphology in A549 cells via the PI3K/Akt–NF-κB–Snail pathway [156, 157]. In COPD models, luteolin alleviated oxidative stress and inflammation via the NOX4-mediated NF-κB signaling pathway [158]. Luteolin also protects against oxidative stress and inflammation in COPD models induced by cigarette smoke by regulating the TRPV1/SIRT6 and CYP2A13/NRF2 pathways [159].

Luteolin is reported to downregulate HIF-1α and NLRP3 inflammasome in neonatal mice [160]. HIF-1α induces CIRP (cold-inducible RNA-binding protein) expression, and extracellular CIRP activates NLRP3 assembly [160]. Luteolin hinders NLRP3 inflammasome-derived caspase-1 activation and release of IL-1β in J774A.1 macrophage exposed to stimuli like nigericin, ATP, or silica crystals [161]. Luteolin treatment decreases the expression of ASC-NLRP3, HSP70, caspase-1, and NF-κB, indicating its capability to modulate NLRP3 inflammasome activity through the HSP70 signaling protein [162]. However, significant data linking luteolin’s effects on NLRP3 to modulate lung fibrosis is currently lacking, indicating the need for further research.

### Kaempferol

Characterized by phenyl rings and four hydroxyl groups, kaempferol is a flavonoid found in various palatable plants like tea, cabbage, kale, beans, broccoli, strawberries, grapes, and numerous medicinal plants such as *Ginkgo biloba* and *Allium fistulosum* [163]. Kaempferol has therapeutic effects on lung cancer by inhibiting xenograft lung cancer models in vivo and activating immune cells. Such inhibitory effects might come from initiating T cells, NK cells, and other immune cells modulated by the gut microbiota [164]. Kaempferol inhibits LPS-induced bronchial EMT in BEAS-2B cells and reduces pro-inflammatory cytokines and pulmonary edema through NF-κB and MAPK signaling pathways in OVA (ovalbumin)-sensitized mice [165]. In the TGF-β1-induced BEAS-2B cells, the secretions of IL-33 and IL-25 and the NOX4-facilitated autophagy were significantly diminished by kaempferol. Further, kaempferol treatment mitigated airway inflammation in the OVA-challenged mice by reducing NOX4-mediated autophagy [166]. Hangqi Liu et al. reported that kaempferol also slows the progression of silica-induced pulmonary fibrosis [167].

Kaempferol also shows promise in inflammatory and immune-related conditions. In acute glaucoma, it reduces retinal ganglion cell death by suppressing caspase-8 and NLRP1/NLRP3 inflammasomes through NF-κB and JNK inhibition [168]. It demonstrated similar protective effects in a retinal ischemia-reperfusion (I/R) model. In corneal allograft transplantation, kaempferol reduced NLRP3/IL-1β axis enhancement and M1 macrophage polarization, inhibiting the NLRP3 inflammasome through autophagy induction [169]. In the collagen-induced arthritis (CIA) mouse model, kaempferol inhibited the NLRP3 inflammasome and NF-κB pathway, reduced IL-1β and other inflammatory markers, and regulated intestinal flora [170]. While kaempferol’s direct role in NLRP3 inhibition in lung fibrosis remains unstudied, its anti-inflammatory, antifibrotic, and antioxidant properties suggest potential benefits.

### Pinocembrin

Pinocembrin is a prominent 5,7-dihydroxy flavanone found in various herbs, fungi, and hive products, including honey, ginger roots, propolis, and wild marjoram [171]. Notably, pinocembrin has progressed to phase II clinical trials for treating ischemic stroke (NCT02059785), attributed to its neuroprotective properties such as reducing ROS, modulating mitochondrial function, protecting the blood-brain barrier, and regulating apoptosis [172].

In a sheep model of BIPF, administration of pinocembrin was associated with significant improvements in lung compliance, reduced inflammation, and decreased immuno-positive T cells within the lung parenchyma [173]. These benefits are primarily attributed to its ability to regulate the TLR4-NF-κB signaling pathway and inhibit the activation of NLRP3 inflammasomes, which are critical in the inflammatory response associated with lung injury [174]. Studies indicate that pinocembrin can attenuate pulmonary edema, reduce histological severity, and decrease inflammatory cell infiltration in LPS and BLM-induced lung injury models, highlighting its promise as a novel candidate for treating inflammatory lung diseases [174].

Additionally, pinocembrin has been shown to restore cognitive function and reduce neuronal apoptosis and hippocampal inflammation in vivo, mainly by inhibiting NLRP3 inflammasome formation, reducing microglial infiltration, and enhancing BNIP3-mediated mitophagy through the JNK-ERK signaling pathway [175]. In vitro studies reinforce these findings, demonstrating pinocembrin’s protective effects against IH-induced cytotoxicity in microglial cells [175]. Furthermore, pinocembrin has been found to suppress oxidized low-density lipoprotein-activated NLRP3 inflammasome and GSDMD-facilitated endothelial cell pyroptosis via an Nrf2-dependent signaling pathway [176]. Pinocembrin’s efficacy in reducing inflammatory markers such as TNF-α, COX-2, IL-1β, iNOS, and MCP-1 has been exhibited in various preclinical and in vitro models, including LPS-induced RAW264.7 and J774A.1 cells [176]. These findings position pinocembrin as a favorable candidate for further exploration in remedying pulmonary inflammatory disorders.

### Chrysin

Chrysin is a bioflavonoid with a distinctive chemical structure comprising one heterocyclic ring (A) and two phenyl rings (B and C). This structural foundation contributes to its pharmacological effects by boosting the body’s antioxidant system by scavenging free radicals, suppressing pro-oxidant enzymes, and chelating redox-active transition-metal ions [177]. In pulmonary health, chrysin has shown substantial promise in mitigating BIPF in rats by reducing mortality rates, improving lung architecture, and lowering hydroxyproline content and TGF-β1 protein expression [178]. Chrysin also reduces inflammatory cell infiltration and lactate dehydrogenase (LDH) activity and alleviates oxidative stress by reducing lipid peroxidation and enhancing antioxidant defenses while mitigating hypoxia in lung tissue by reducing hypoxia-inducible factor 1-alpha levels [178].

Chrysin’s ability to inhibit NLRP3 inflammasome activation further highlights its therapeutic potential, particularly in conditions like hepatic fibrosis [179] and synovial inflammation [180]. Chrysin activates AMPK phosphorylation in hepatic fibrosis and inhibits NLRP3 inflammasome activation, reducing inflammatory injury and pyroptosis [179]. Similarly, in models of synovial inflammation, chrysin alleviates pain and synovitis by inhibiting the PERK/TXNIP/NLRP3 signaling pathway [180]. Moreover, chrysin protects against iron overload-induced hepatocellular damage by reducing oxidative damage biomarkers and suppressing pro-inflammatory cytokines. It enhances the formation of redox and inflammation-regulating proteins such as SIRT1, PPARγ, and the antioxidant protein Nrf2 while suppressing NLRP3 inflammasome assembly and NF-κB acetylation [179].

Chrysin’s broad spectrum of pharmacological activities also includes anti-allergic and anti-proliferative properties. It has effectively prevented LPS-induced ALI in mice by suppressing the IRE1α/TXNIP/NLRP3 pathway, which reduces pulmonary edema, vascular leakage, inflammatory cytokines, and MPO activity while improving redox homeostasis [181]. Additionally, chrysin-loaded PLGA (poly(lactic-co-glycolic acid)) nanoparticles have shown promise in attenuating OVA-activated allergic asthma by altering the TLR/NF-κB/NLRP3 axis [182]. This modulation reduces airway inflammation, decreases pro-inflammatory cytokine levels, and stops NLRP3 inflammasome assembly [182]. These findings underscore chrysin’s potential as a therapeutic agent in managing allergic respiratory conditions such as asthma, highlighting its multifaceted role in regulating inflammatory pathways, thereby treating IPF and various interstitial lung diseases.

### Diosmin

Diosmin is a naturally occurring flavone glycoside prominently found in citrus fruits, with broad pharmacological activities [183]. Upon ingestion, it is hydrolyzed by intestinal microflora into diosmetin, which plays a crucial role in treating venous diseases, particularly chronic venous insufficiency, and as a protective agent for blood vessels, influencing venous wall tone, permeability, lymphatic drainage, and reducing inflammation and oxidative stress [183, 184]. In experimental studies, diosmin has shown significant protective effects against pulmonary and renal fibrosis. In a paraquat-induced lung injury model, diosmin increased glutathione levels, decreased hydroxyproline content, and reduced tissue damage [185]. In a unilateral ureteral obstruction (UUO) mouse model, diosmin attenuated renal fibrosis and lowered inflammatory cytokine levels by inhibiting NF-κB P65 through SIRT3 activation [186]. Diosmin also exhibits promise in treating LPS-induced ALI in mice, significantly reducing inflammatory cells, pro-inflammatory cytokines (IL-6 and IL-2), and lung edema [187].

In neurological studies, diosmin suppresses inflammation, oxidative damage, and neuronal degeneration. It improves cell viability in neuron and microglia co-culture systems by inhibiting the NLRP3 inflammasome and stimulating SIRT1 signaling [188]. Diosmin also mitigates high glucose-induced stress in HK-2 cells through the PI3K/AKT pathway, reducing key markers like IL-1β and NLRP3 [189]. Additionally, diosmin has protective effects in mice’s prefrontal cortex (PFC), alleviating LPS-induced depressive-like behaviors by reducing pro-inflammatory cytokines, microglial activation, and NLRP3 inflammasome expression [190]. However, no studies have been found to link the therapeutic effects of diosmin in IPF through NLRP3 inflammasomes.

### Nobiletin

Nobiletin, a poly-methoxy flavonoid predominantly found in citrus peels, exhibits a broad spectrum of pharmacological activities, as listed in Table 2 [191]. At the molecular level, nobiletin modulates several key signaling pathways, including PKA/ERK/MEK/CREB, NF-κB, AMPK, PPAR-α, and PI3K/AKT [191]. Its antifibrotic effects are particularly notable, as it inhibits NLRP3-associated inflammation and suppresses the PI3K/AKT/mTOR pathway, thereby reducing fibrotic processes in amiodarone-induced pulmonary fibrosis [192]. The activation of AMPK-mTOR-mediated autophagy further enhances its antifibrotic capabilities [192]. In non-small-cell lung cancer (NSCLC), nobiletin inhibits cancer cell proliferation and induces apoptosis by modulating the WNT/β-catenin and miR-15-5p/β-catenin signaling pathways [193]. In other respiratory conditions such as COPD and ALI, nobiletin mitigates inflammation-related pathways, including TNF, MAPK, and PI3K-AKT [194]. It also inhibits ferroptosis through the p53/SLC7A11 pathway [195].

Additionally, nobiletin has shown promise in neuroprotection, particularly in LPS-induced neuroinflammation models. It improves behavioral deficits, promotes autophagy, and attenuates NLRP3 inflammasome activation via the AMPK pathway [196]. Nobiletin also plays a crucial role in cardiovascular health by activating PINK1/Parkin-mediated mitophagy, which helps reduce lipid uptake, intra-arterial lipid accumulation, and macrophage infiltration, thereby exerting anti-atherosclerosis effects [197]. In metabolic health, nobiletin has been shown to alleviate palmitic acid-induced lipotoxicity in AML‑12 hepatic cells by suppressing NLRP3 inflammasome assembly in a SIRT1-dependent process, leading to reduced expression of inflammatory cytokines (IL-18 and IL-1β) [198]. Therefore, nobiletin can be considered a potential molecule in treating pulmonary fibrosis by inhibiting the NLRP3 pathway.

## Future Perspectives and Limitations

This review highlights the substantial promise of flavonoids in modulating the NLRP3 inflammasomes, which are crucial mediators of the inflammatory response and fibrosis in IPF. Since the NLRP3 inflammasome is a significant module in the inflammatory cascade leading to tissue damage and lung fibrosis, targeting this pathway is an innovative and attractive therapeutic strategy. With only two drugs currently approved for treating IPF, expanding the therapeutic landscape by exploring flavonoids offers a novel and promising direction for future research and clinical development.

In IPF, certain flavonoids have shown specific inhibitory effects on NLRP3 inflammasome activation, directly impacting the fibrotic process. Catechins, pinocembrin, chrysin, and nobiletin have demonstrated anti-inflammatory and antifibrotic properties by targeting the NLRP3 inflammasome pathway. On the other hand, flavonoids like kaempferol and diosmin exhibit beneficial effects in lung fibrosis but do not directly act via NLRP3 inhibition. Instead, they exert their therapeutic effects through other pathways, such as inhibiting MAPKs and NF-κB, reducing oxidative stress, and modulation of inflammatory cytokines. Hence, future research should prioritize identifying and validating other potent flavonoids that can specifically target NLRP3 inflammasomes and clarify their mechanisms of action.

Utilizing advanced in silico technologies and high-throughput virtual screening (HTVS) methods could accelerate the discovery of flavonoids with high specificity and efficacy against NLRP3. Additionally, expanding the repository of cell culture and preclinical studies is crucial to establishing a more comprehensive understanding of these compounds’ pharmacodynamics, pharmacokinetics, and bioavailability in human models. In addition to preclinical validation, clinical trials will be pivotal in translating the promising preclinical results into clinical practice. These trials should rigorously assess the safety, efficacy, and optimal dosing regimens of flavonoids for IPF patients. Flavonoids may not serve as a replacement for existing IPF therapies; however, their potential to complement current treatments in a combinational approach presents a promising avenue for further research. Studies have highlighted that combinational treatments may enhance therapeutic efficacy or reduce adverse effects, thus offering a more comprehensive approach to managing the disease [199].

Despite the promising findings presented in this review, limitations in the current research need to be acknowledged. Many studies are still in the preclinical stage, and there is a lack of robust pharmacokinetic and clinical data to confirm the therapeutic potential of these flavonoids [200]. The bioavailability and stability of flavonoids remain concerns, as they may require formulation strategies, such as nano-delivery systems, to ensure adequate absorption and therapeutic concentrations in the desired tissues [201]. Additionally, the long-term safety profiles of flavonoids in IPF patients need thorough investigation, especially regarding their potential interactions with other medications.

## Conclusion

IPF remains a life-threatening condition with limited therapeutic alternatives, emphasizing the need for innovative treatments. This review underscores the potential of flavonoids as modulators of the NLRP3 inflammasome, a critical driver of inflammation and fibrosis in IPF. Compounds like catechins, chrysin, pinocembrin, and nobiletin have shown promising antifibrotic and anti-inflammatory effects, directly targeting NLRP3, while others like kaempferol and diosmin exhibit therapeutic benefits through alternative pathways. These findings highlight flavonoids’ multifaceted role in mitigating fibrosis and inflammation, making them attractive candidates for future therapeutic development.

However, significant challenges remain in translating these preclinical outcomes into clinical reality. Issues such as poor bioavailability and the lack of robust clinical data must be addressed through innovative nano-delivery formulations, advanced computational screening technologies, and well-designed clinical trials. Moreover, exploring the combinational potential of flavonoids with existing therapies could further enhance treatment efficacy and tolerability. By bridging these gaps, flavonoids have the potential to revolutionize IPF treatment, offering new hope to patients and redefining the therapeutic landscape of pulmonary fibrosis.

## Data Availability

No datasets were generated or analysed during the current study.
